# Meta-analysis of sources and transmission pathways of *Apis mellifera* (Hymenoptera: Apidae) microbiota based on 16S sequencing data

**DOI:** 10.1093/jisesa/ieaf093

**Published:** 2025-12-23

**Authors:** Artur Asadullin, Grigory Kashchenko, Alexander Klochev, Amir Taldaev, Leonid Adonin, Daniil Smutin

**Affiliations:** Federal State Budget-Financed Educational Institution of Higher Education, The Bonch-Bruevich Saint-Petersburg State University of Telecommunications, St. Petersburg, Russia; Department of Genetics and Biotechnology, St. Petersburg State University, St. Petersburg, Russia; Sirius University of Science and Technology, Sochi, Russia; Russian State Agrarian University–Moscow Timiryazev Agricultural Academy, Moscow, Russia; Department of Genetics and Biotechnology, St. Petersburg State University, St. Petersburg, Russia; Institute of Biomedical Chemistry, Moscow, Russia; Research Center for Molecular Mechanisms of Aging and Age-Related Diseases, Moscow Center for Advanced Studies, Moscow, Russia; Shemyakin-Ovchinnikov Institute of Bioorganic Chemistry, Moscow, Russia; Federal State Budget-Financed Educational Institution of Higher Education, The Bonch-Bruevich Saint-Petersburg State University of Telecommunications, St. Petersburg, Russia; Federal State Budget-Financed Educational Institution of Higher Education, The Bonch-Bruevich Saint-Petersburg State University of Telecommunications, St. Petersburg, Russia; Institute of Biomedical Chemistry, Moscow, Russia; Institute of the Applied Computer Science, ITMO University, St. Petersburg, Russia

**Keywords:** bacterial transfer, hive microbiome, meta-analysis, structural equation modeling (SEM)

## Abstract

This study investigates the mechanisms governing the formation and transfer of microbial communities associated with the honey bee (*Apis mellifera* L.) superorganism, focusing on the interplay between plant, in-hive, and bee environments. By analyzing 16S rRNA sequencing data from multiple public datasets through bioinformatics and statistical modeling, we characterized the structure and transmission pathways of these microbiota. Our analysis reveals that each environment hosts a distinct and specialized microbial community, with significant barriers to free microbial exchange. Alpha and beta-diversity analyses confirmed the uniqueness of the bee gut microbiota and the mixed, intermediate nature of the honey microbiome. Structural equation modeling identified that direct microbial transfer from plants to bees is negligible. Instead, honey serves as an obligate intermediary and selective filter, with microorganisms transitioning from plants to honey before a lower-probability transfer to bees occurs. Furthermore, we identified key bacterial taxa, including *Apilactobacillus kunkeei*, *Acinetobacter*, and *Pseudomonas*, that potentially act as generalists capable of persisting across multiple environments. These findings underscore the possibility of the selective bacterial transfer between hives, which may play roles in both pathogens transfer and maintaining hive microbiome stability.

## Introduction

The microbiota of a hive of honey bees (*Apis mellifera* L.; Hymenoptera: Apidae) represent a complex assembly of microbial communities inhabiting both various hive environments and the bees themselves. These microorganisms play a crucial role in maintaining immunity, influencing metabolic processes, and contributing to the “maturation” of bee bread and honey, ultimately affecting bee behavior (Kwong and Moran 2016, Iqbal et al. 2022, Smutin et al. 2022, Tiusanen et al. 2024, Anderson and Mott 2023). Microbial communities can enter the hive through contact with the external environment—plants and soil—or be transmitted horizontally via interactions among colony members, hive pests, and other hive contents, as well as vertically through inheritance (Miller et al. 2019, Anderson et al. 2023). Despite the apparent simplicity of these transmission pathways, distinguishing between strictly horizontal and vertical transmission is challenging due to the significant species diversity and the multitude of ecological niches occupied by these microorganisms. The presence of bacteria identified as the same species through metagenomic methods does not necessarily imply that all such bacteria are transmitted between these environments. To confirm or refute this hypothesis, controlled model experiments are required. Indirect evidence can be obtained by considering ecological species criteria: if microbial interactions persist across different environments, it is more likely that the respective species overlap, making their transmission patterns a rational subject of investigation.

Previous studies have described potential transmission pathways and microbiome connectivity based on various sequencing results. However, genetic diversity data on hive microbiota, such as *Apilactobacillus kunkeei*, indicate that in some cases, the unrestricted transmission of this bacterium—even between different hive environments—is hindered due to its adaptation to specific ecological conditions (Neveling et al. 2012). On the other hand, some researchers propose that observed habitat specialization does not preclude the possibility of *A. kunkeei* being indirectly transmitted between hives via plants. Understanding these processes is crucial, as the observed patterns point to an unexplored functional role of this bacterium, which may have broader implications, including its impact on economic aspects of beekeeping—particularly on hive productivity and stability.

This study analyzes publicly available 16S rRNA sequencing datasets to characterize the plant and bee microbiome. The first dataset (Prado et al. 2020, Tiusanen et al. 2024) examines microbial transitions along the bee–honey–plant pathway. The second dataset profiles the microbiomes of social (*A. mellifera*) and solitary (*Osmia bicornis*) bees pollinating *Brassica napus*. Our primary objective was to investigate the transfer of microflora between plants and their pollinators.

Approximately one-quarter of the bacterial community biodiversity in bees overlaps with that of plants (Tiusanen et al. 2024). We hypothesized that distinct groups of microorganisms are transferred. While most microbiome members in environments such as the honey bee gut, in-hive substances (honey, corbicular pollen), and plants are specialists, a subset may persist outside their native communities. Through this persistence, some microorganisms can colonize and influence the formation and succession of novel environments. Furthermore, certain cosmopolitan microorganisms might proliferate across different environments during specific community stages, potentially playing a role in controlling the maturation of these communities.

## Materials and Methods of Research

### Data Libraries Used

For microbiota analysis, we utilized 16S rRNA sequences from the publicly available BioProject PRJNA930592 and PRJEB31847 datasets. (Prado et al. 2020, Tiusanen et al. 2024). The sequences of PRJNA930592 were obtained from homogenized samples of nurse and forager bees of *A. mellifera*, freshly collected honey, and inflorescences of abundantly flowering plants. Sampling was conducted during the second decade of each summer month in 2021 near apiaries in South Finland. A more detailed description of the study locations and the methodology can be found in Tiusanen et al. (2024).

PRJEB31847 data were a result of the model experiment. From plants were independently collected the seed, nectar and pollen material as well as whole bees (*A. mellifera* and *O. bicornis*, both cuticular assemblages and the full bodies). More details are provided in the study Prado et al. (2020).

### Data Processing and Analysis

Microbial sequence data were processed using the QIIME 2 pipeline. The initial steps included quality filtering, denoising, and taxonomic assignment. Upon import, sequences were demultiplexed using the *demux* plugin (Hamady et al. 2008), followed by denoising with *DADA2* (Callahan et al. 2016) to infer amplicon sequence variants. The *DADA2* parameters were set as follows: —p-max-ee 2, —p-trunc-q 2, and —p-trim-left 20. Taxonomic classification was performed using a pre-trained Naïve Bayesian classifier, based on the SILVA database (Quast et al. 2013), implemented via the *sklearn* algorithm in QIIME 2 (Bokulich et al. 2018, Robeson et al. 2021).

Statistical analysis was performed using the R programming language (version 4.4.1+) (R Core Team 2024) alongside with packages *phyloseq* (McMurdie and Holmes 2013) and *microbiome* (Lahti and Shetty 2012). All nondeterministic analyses were conducted under the controlled seed = 123. Samples were rarefied at the 1,000 reads depth for the diversity analysis, and 10,000 reads for the SEM and network analysis. Diversity analysis and ordinations were performed with the application of the *vegan* package, which was used for calculating ecological indices and performing multidimensional scaling (Oksanen et al. 2024). Structural equation modeling (SEM) analysis between ecological and taxonomic characteristics was conducted using the *lavaan* package (Rosseel 2012). We reconstructed networks of SparCC correlations with the threshold 0.3 using NetCoMi package (Yoon et al. 2019, Peschel et al. 2021). An indicator was defined as zOTU that present in at least 10% of the samples within a given group and exhibited the highest mean abundance in that group compared to all other groups. Visualization was carried out using the *ggplot2* (Wickham 2016) and *ggpubr* packages (Kassambara 2023). Graphics were additionally extended with *ggviolinbox* (Smutin 2025), *ggtext* (Wilke and Wiernik 2022), and *ggthemes* (Arnold 2024).

## Results

The analysis was conducted on data from 2 distinct bioprojects (PRJNA930592 and PRJEB31847), which were processed separately. A detailed summary of the sequence filtration statistics is provided in Supplementary Table S1. Following preprocessing of the PRJNA930592 dataset, rarefaction at a depth of 1,000 reads per sample yielded 791 samples comprising 3,369 zero-radius operational taxonomic units (zOTUs). When rarefied to a depth of 10,000 reads, the same dataset contained 556 samples and 3,317 zOTUs. A comparable preprocessing workflow applied to the PRJEB31847 dataset resulted in 198 samples (3,132 zOTUs) after rarefaction to 1,000 reads, and 196 samples (6,590 zOTUs) after rarefaction to 10,000 reads. Sample metadata, including data classes, were extracted from the corresponding SRA entries (Supplementary Fig. S1).

**Fig. 1.**
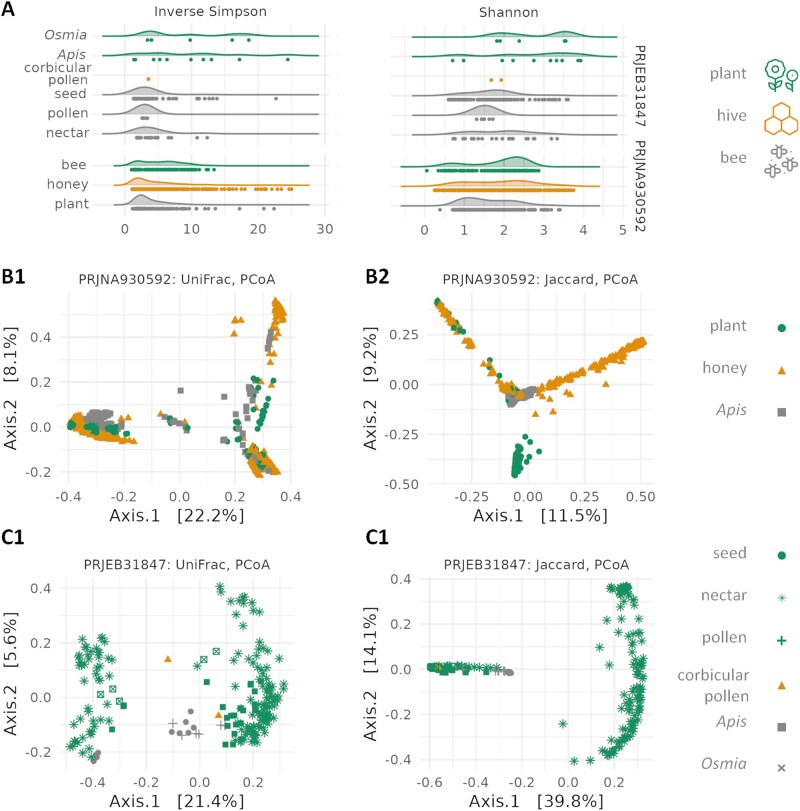
Microbial community diversity analysis. A) Raincloud plots depicting variations in alpha diversity, as measured by the Inverse Simpson and Shannon indices. Samples are grouped by environment and bioproject. Principal Coordinate Analysis (PCoA) ordination plots illustrating beta diversity for the (B) PRJNA930592 and (C) PRJEB31847 datasets. Plots are based on unweighted UniFrac (B1, C1) and Jaccard (B2, C2) distance matrices. Data points are colored by hive of origin or experimental environment and shaped by source material.

Microbial community diversity, as measured by the Shannon and Inverse Simpson indices, varied significantly across different environments (Fig. 1). In the PRJNA930592 dataset, the honey bee and honey communities exhibited significantly higher Shannon diversity than the plant microbiome (*P *< 0.001; Fig. 1A, Supplementary Table S2). Similarly, the Inverse Simpson index was significantly higher in bee communities compared to both honey and plant communities (*P *< 0.005, Supplementary Table S2), while no significant difference was observed between honey and plant communities themselves (*P *≈ 0.22, Supplementary Table S2).

Analysis of the PRJEB31847 dataset revealed that the microbiomes of social (*A. mellifera*) and solitary (*O. bicornis*) bees were not significantly different in terms of either diversity index (*P *≈ 0.8, Supplementary Table S2). A lack of significant differentiation was also found among the various plant compartments (seeds, pollen, nectar; *P*-values ranging from 0.1 to 0.8, Supplementary Table S2). Consistent with the first dataset, all bee-associated microbiomes demonstrated significantly higher alpha diversity than those of plant-associated samples.

Beta-diversity analysis revealed clear separation between sample groups from different environments (Fig. 1B and C). While sample type was a primary driver of clustering, data points within the PRJNA930592 dataset also grouped according to other variables (Fig. 1B). To identify the sources of this variation, we performed an Non-metric multidimensional scaling (NMDS) ordination and fitted environmental variables to the result (Supplementary Fig. S2A). Sampling year (*R*^2^ = 0.20) and environment (*R*^2^ = 0.10) had the strongest significant influence on the ordination. In contrast, the summer sampling month did not have a significant effect on the Bray–Curtis-based NMDS after rarefaction (*R*^2^ = 0.01; all Pr(*>F*) = 0.001, Supplementary Table S3).

PerMANOVA analysis on both UniFrac and Jaccard distances confirmed the dominant influence of environment over potential temporal biases (Jaccard: environment *R*^2^ = 0.09, year *R*^2^ = 0.02, month *R*^2^ = 0.01, all Pr(*>F*) = 0.001; UniFrac: environment *R*^2^ = 0.07, year *R*^2^ = 0.02, month *R*^2^ = 0.01, all Pr(*>F*) = 0.001; Supplementary Table S4).

Honey samples formed 2 distinct clusters in the Bray–Curtis NMDS and Jaccard PCoA ordinations: One group clustered within other sample types, and a second group was distinctly separate. The presence and abundance of *A. kunkeei* was the only species-level factor that significantly differentiated these 2 groups (Wilcoxon test, *W* = 5,058.5, FDR-adjusted *P *≈ 10^−13^), indicating its key role in structuring the honey microbiome and influencing alpha-diversity (Supplementary Fig. S3). All honey samples marked with an asterisk (*) in Supplementary Fig. S2A, which lacked *A. kunkeei*, exhibited significantly lower Shannon and Inverse Simpson indices. Furthermore, intermediate abundances of *A. kunkeei* were associated with the highest Shannon diversity values among honey samples (Supplementary Fig. S3C). The microbial community of bees was the most distinct, predominantly composed of specialized anaerobic or microaerobic species. Honey microbiota occupied an intermediate position, reflecting its mixed origin and influence on in-hive fermentation processes.

**Fig. 2.**
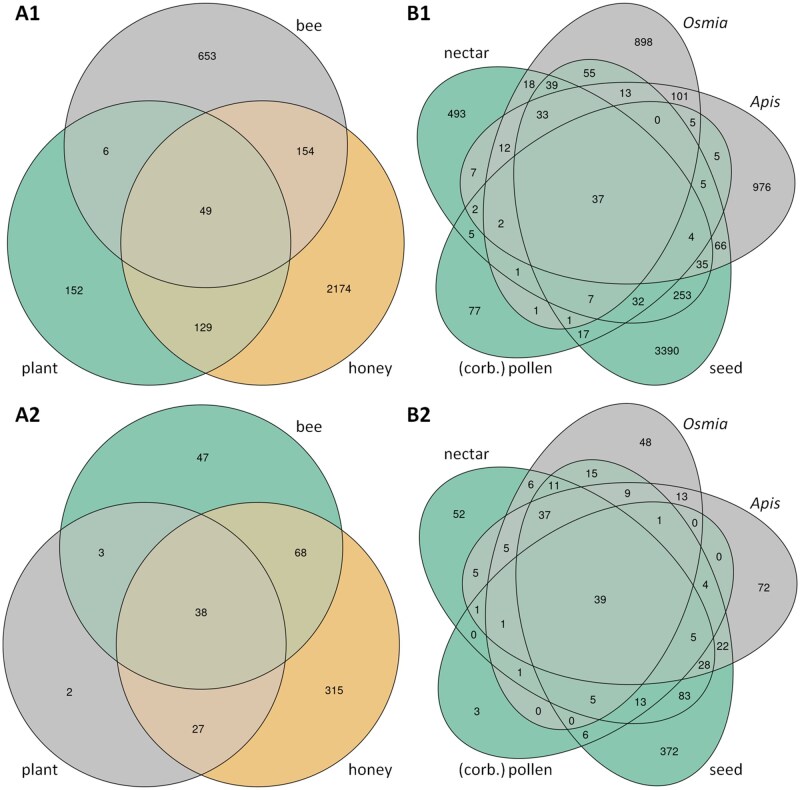
Distribution of zOTUs and species across environments. Venn diagrams show the overlap of (A) zOTUs and (B) species detected in different environments for the PRJNA930592 (A1, B1) and PRJEB31847 (A2, B2) datasets. A list of the “generalist” zOTUs identified from this analysis is provided in Supplementary Fig. S4.

Beta-diversity analysis of the PRJEB31847 dataset revealed pronounced differences between plant and bee microbiomes (Fig. 1C, Supplementary Fig. S2B). This separation was strongly influenced by environment, as indicated by PerMANOVA results (Jaccard *R*^2^ = 0.31; UniFrac *R*^2^ = 0.07; all Pr(*>F*) = 0.001; Supplementary Table S4). The strong differentiation may be a consequence of sampling less sugar-rich plant compartments, such as pollen and seeds, in this dataset. Furthermore, significant differences in beta-diversity were observed between the microbiomes of *A. mellifera* and *O. bicornis* (UniFrac: *R*^2^ = 0.13, Pr(*>F*) = 0.014; Jaccard: *R*^2^ = 0.18, Pr(*>F*) = 0.001; Supplementary Table S4). To identify potential microbial transfer groups, we analyzed the overlap of zOTUs and species between environments in both datasets (Fig. 2). A key finding was that microbial taxa detected in both plants and bees were almost invariably present in honey (Fig. 2A); only 6 zOTUs (3 species) were common to plants and bees but absent in honey. This pattern supports the hypothesis that honey acts as an obligate intermediate environment for microbial transfer from plants to bees. It suggests that microorganisms capable of colonizing the bee gut may first undergo a selection stage in honey, where they encounter conditions such as high osmolarity and microbial metabolites.

Plant-associated environments (nectar, seeds, pollen) contained fewer microbial groups unique to plants compared to groups shared with bees (Fig. 2B). Specifically, 32 zOTUs (13 groups at the species level) were unique to plant compartments, whereas 37 zOTUs (39 species) were found across all environments (bees, honey, and plants). An additional 48 zOTUs (49 species) were shared between all plant environments and at least 1 bee-associated environment. This distribution supports the distinction between environment-specific specialists and potential generalist taxa capable of transfer.

We further identified these putative generalist taxa by analyzing zOTUs shared between the 2 datasets (Supplementary Fig. S4). This group included well-known core gut bacteria (*Frischella*, *Snodgrassella*, *Gilliamella*, and *Lactobacillus*) alongside aerobic hive community members such as *A. kunkeei*, *Acinetobacter* sp., and *Pseudomonas* sp. The presence of core gut taxa in aerobic environments (eg plants, hive surfaces) supports the possibility of their transfer. The aerobic species identified may represent transfer groups adapted to plant, hive, and bee environments. Alternatively, they may indicate early speciation events, where similar zOTUs occur independently in different environments without active transfer.

**Fig. 3.**
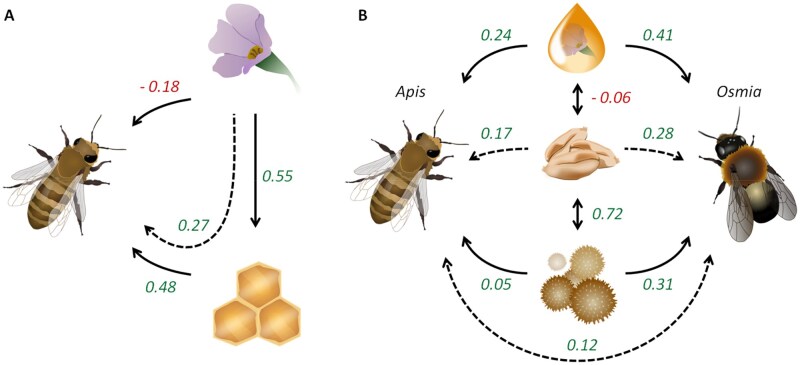
SEM of microbial transfer pathways. A) Significant direct and indirect transfer paths between *Apis mellifera*, plants, and honey, inferred from the PRJNA930592 dataset. B) Significant transfer paths between bees (*Apis mellifera* and *Osmia bicornis*) and plant environments (nectar, seeds, pollen). Dotted lines represent indirect transfer paths. All paths shown are statistically significant. Detailed model selection criteria are provided in Supplementary Figs S5 (PRJNA930592) and S6 (PRJEB31847).

Putative microbial transfer pathways were investigated using SEM. Details on model selection and path coefficients are provided in Supplementary Figs S5 and S6. The analysis revealed that direct microbiota transfer from plants to bees is negligible (Fig. 3A). Instead, plant microbiota exhibit a high probability of transition into honey, followed by a lower-probability transfer from honey to bees. This suggests an indirect pathway whereby bees inherit a limited diversity of plant microbiota via honey.

**Fig. 4.**
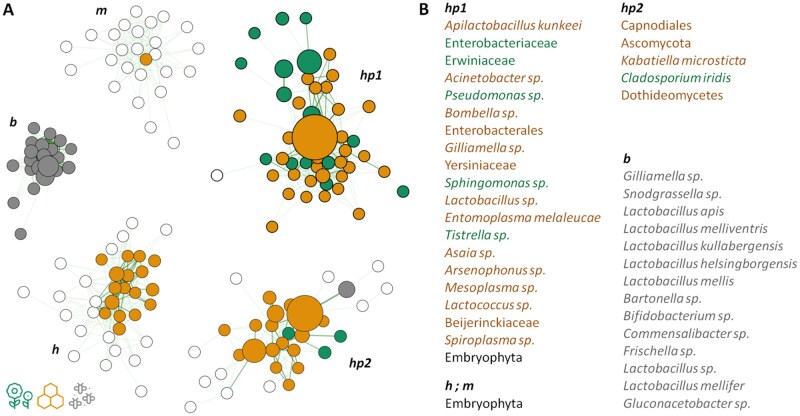
Co-occurrence network analysis of the PRJNA930592 dataset. A) A SparCC correlation network constructed with the NetCoMi package (correlation threshold |*r*| > 0.3; insignificant correlations omitted). Node size corresponds to the normalized mean abundance of the zOTU across samples. Node color represents the environment in which the zOTU was indicative. B) Taxonomic classification of the features, ordered by their normalized mean abundance and colored according to their indicative environment.

**Fig. 5.**
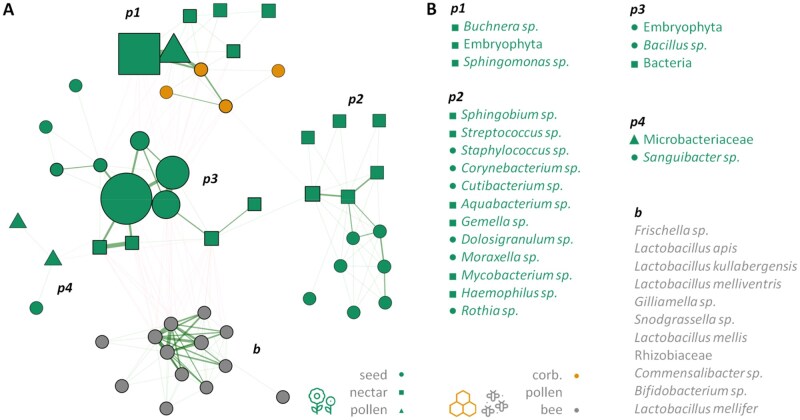
Co-occurrence network analysis of the PRJEB31847 dataset. A) A SparCC correlation network constructed with the NetCoMi package (correlation threshold |*r*| > 0.3; insignificant correlations omitted). Node size corresponds to the normalized mean abundance of the zOTU across samples. Node color and shape represent the environment in which the zOTU was indicative. B) Taxonomic classification of the features, ordered by their normalized mean abundance and colored according to their indicative environment.

In the PRJEB31847 dataset, the strongest transfer path was from nectar to bees (Fig. 3B). However, a secondary pathway was observed between pollen and seeds, mediated by bees, indicating a potential role for bees in influencing seed microbiome colonization. The higher transfer coefficients observed between *Osmia* and plants compared to *Apis* and plants may be a consequence of experimental design; the higher abundance of *Apis* pollinators could dilute the bacterial titer transferred per interaction.

To further identify potential transfer groups, we reconstructed their ecological relationships using co-occurrence networks. In the PRJNA930592 network, the largest clusters corresponded to different indicator groups within honey (Fig. 4) (hp1, hp2). The most isolated cluster comprised core honey bee gut members (b), which represent the only fully anaerobic group. Two other, less-connected clusters contained either unclassified zOTUs or those predicted to be of plant origin (h, m).

Similarly, the largest correlation clusters in the PRJEB31847 network consisted of different plant indicators (Fig. 5). In contrast, nonindicator species showed few significant correlations, potentially due to their smaller group sizes. This network also revealed more negative correlations, including antagonistic relationships between bee gut microbiome indicators and plant microbiomes that were absent in the first dataset. This contrast further underscores the role of honey as a buffer and intermediary in microbiome transfer. Under the conditions of the second experiment, certain bee gut microbes (eg *Bifidobacterium*, *Commensalibacter*) that were present in all environments in the first dataset were absent from plants. This difference may also stem from the inclusion of 2 bee species with distinct known microbiomes (Tsadila et al. 2023).

## Discussion

The results highlight the significant impact of environmental factors on the structure and diversity of microbial communities within the hive. Specific levels of humidity, moderated daily temperature fluctuations, and the “selectivity” of the hive environment create a unique microclimate that shapes microbial community composition. Compared to external conditions, the hive offers fewer but more stable niches, influencing the dominance of certain microbial groups such as Lactobacillus, Bifidobacterium, Frischella, and Gilliamella, as well as osmotolerant yeasts characteristic of honey. The first 2 genera not only contribute to the lowered pH values of various hive environments (which may serve a protective function against certain pathogens) but are also involved in the metabolism of di- and monosaccharides present in honey. Several studies have demonstrated the presence of plant pathogenic microbiota within the microbial communities of honey bees and the hive (Neveling et al. 2012, Kwong and Moran 2016, Thamm et al. 2023, Anderson and Copeland 2024, Anderson and Mott 2023).

Saccharolytic microorganisms, isolated from plant material and performing similar functions within the bee colony, dominate all environments except the intra-hive space (Thamm et al. 2023). Their transfer from the external environment occurs through the collection of pollen and nectar.

Thus, the microbial profiles of bee-derived products (such as bee bread and honey), shaped by the activity of osmotolerant microorganisms and lactic acid bacteria (LAB group), make the survival of inherently pathogenic microbiota nearly impossible even at early stages of maturation. The resulting conditions lead to a reduction in microbial diversity in these environments (Anderson and Mott 2023). The processes causing a decrease in bacterial abundance may result in a low likelihood of effective microbiota transfer from food resources to the native microbiomes of bees. Here, despite a significant regression coefficient characterizing the quantitative transfer of microbiota from one environment to another (between plants—pollen and nectar—and honey), there is a clear reduction in both diversity and abundance of microorganisms due to the “extremeness” of the conditions created by this very community.

The in-hive environment, functioning as an obligatory intermediate medium in the transfer of microbiota from plant sources to bee organisms, suggests that a significant proportion of microorganisms potentially capable of colonizing the bee gut undergo a selection stage in honey. In this environment, they are exposed to specific conditions, such as high osmolarity and the presence of metabolites produced by other microorganisms, which may determine their viability and successful transmission into the bee microbiome.

These findings indicate a complex and multifaceted nature of microbial transfer. The reason also may lie in dietary variations. The primary diet of foragers during the summer consists of pollen and nectar, rather than honey produced in the current season (Nicolson 2011, Vaudo et al. 2024). Only in rare cases, when environmental changes lead to a reduction in the colony’s food resources, does active consumption of honey begin (Blaschon et al. 1999, Karbassioon et al. 2023).

Our analysis provides new insights into the specific bacterial groups involved in cross-environmental transfer. We identified 2 potential colonization pathways for bees. The first involves core gut species that may only persist transiently on external surfaces; these microbes likely colonize newly emerged bees through social interactions or via hive environments like honey. The second pathway involves bacteria that actively colonize bees during their maturation. Hives appear to acquire *A. kunkeei* from various sources, and individual hives may harbor distinct strains originating from different reservoirs, including those transferred from plants (Maeno et al. 2016, 2021). A similar pattern may apply to other generalist taxa, such as *Acinetobacter* (Kim et al. 2014) and *Pseudomonas* (Tsadila et al. 2023), which were also detected across all environments studied.

The differences in bacterial transfer pathways observed between *Apis* and *Osmia* species, as revealed by SEM, could be attributable to experimental design or reflect genuine biological distinctions. We hypothesize that the most significant plant-to-bee transfer may occur via aerobic bee compartments, such as the midgut or the body surface. While the midgut hosts specialized members (eg *A. kunkeei*, *Bifidobacterium asteroides*), the cuticular surface of *Apis* is notably less colonized than that of *Osmia*. However, the cuticular communities of both forager species are predominantly composed of microbes atypical of hive environments, including generalists like *Acinetobacter* and *Pseudomonas* (Kashchenko et al. 2025). This supports the hypothesis of microbial transfer coupled with adaptation to multiple habitats. From an evolutionary perspective, the higher potential cost of pathogen transfer for eusocial bees like *Apis*, compared to solitary bees like *Osmia*, may explain the observed differences in cuticular bacterial loads. This differential colonization likely has significant implications for the efficiency and dynamics of bacterial transfer between plants, the hive environment, and the bees themselves.

The colonization of microorganisms on the surfaces and within the bodies of bees occurs differently. Microbiota native to the bee gut, entering the organism from the external environment, has a much higher chance of survival under optimal conditions. The inheritance of plant microbiota by bees through honey suggests that food-associated microbiota may play a role in maintaining and shaping the bacterial profile of the bee colony. As a result of trophallaxis and allogrooming occurring among all members of the hive, microbial succession takes place, not only sustaining but also complementing existing bacterial profiles.

The work has some technical limitations. Despite choosing a fairly low annotation level (zOTU), the actual classification level may vary from strains to genera for different groups, as zOTU will combine reads from taxa that do not differ at the level of the studied short 16S rRNA gene fragments. Also, when comparing data from the 2 experiments used at the zOTU level, it is impossible to do so, which forces us to rely on their taxonomic annotations. Despite the availability of the excellent BEExact annotation database, which specializes in the microflora of *A. mellifera* bees, it cannot be used for annotation in projects that combine data on the intestinal microflora of bees with data on hive and plant communities, which is why some annotations remain problematic. This is most acute for Lactobacillus annotation: This large genus includes representatives of the gut microbiome (along with Bombilactobacillus), as well as the microflora of honey at least at some stages, and nectar communities. It is effectively impossible to determine the actual transfer of these species between communities. This illustrates that due to methodological constraints sometimes we cannot divide species cosmopolitism and early stages of sympatric speciation through niche partitioning.

The study of microbiota in bees, honey, and plants revealed key patterns in the structure and transmission of microbial communities. Analysis of alpha and beta diversity demonstrated the uniqueness of microbial communities in each environment, indicating the presence of ecological barriers that limit the free transfer of microorganisms. The microbiota of bees were found to be the most distinct, highlighting its specialization, while the microbiota of honey exhibited a mixed origin, reflecting influences from both plants and intra-hive fermentation processes.

Structural equation modeling analysis confirmed that direct transfer of microbiota from plants to bees is virtually absent, but indirect influence through honey is possible, albeit on a limited scale. This underscores the complexity of microbial community interactions and the importance of environmental factors, such as bee diet and hive conditions. The microbial profiles of honey and bee bread are shaped by microorganisms producing lactic acid and short-chain fatty acids, creating unfavorable conditions for pathogens. This study supports the established hypothesis of the aerobic bacterial hive community significance, delineating its key organisms, succession, and transmission. The stability of the honey bee colony microbiota across generations is partially maintained through social behaviors such as trophallaxis and allogrooming, which facilitate the transfer and establishment of microbial communities among nestmates. While the gut microbiota is often the primary focus, our findings demonstrate the potential of the other horizontal microbial transfer way—between different hive environments and elucidate the pathways involved.

## Supplementary Material

ieaf093_Supplementary_Data
