# Supplementary material for: Meta-analysis of sources and transmission pathways of Apis mellifera (Hymenoptera: Apidae) microbiota based on 16S sequencing data
Source: J Insect Sci. 2025 Dec 23;25(6):ieaf093. doi: 10.1093/jisesa/ieaf093 (PMC12726919; doi:10.1093/jisesa/ieaf093)

**Supplementary Table S1.** Data processing statistics.

| **Parameter** | **Stage** | | **PRJNA930592** | | **PRJEB31847** | |
| --- | --- | --- | --- | --- | --- | --- |
| **Total number of reads** | Raw | | 21 494 610 | | 14 155 519 | |
|  | Filtered | | 16 179 926 | | 13 319 550 | |
|  | Denoized | | 16 079 308 | | 13 262 385 | |
|  | Merged | | 14 017 777 | | 12 919 646 | |
|  | Non-chimeric | | 13 248 704 | **62%** | 11945073 | **84%** |
| **Number of samples** | Raw | | 846 | | 198 | |
|  | After preprocessing | | 846 | | 198 | |
|  | Rarefied | 1 000 | 791 | **93%** | 198 | **100%** |
|  |  | 10 000 | 556 | **66%** | 196 | **99%** |
| **Unique zOTUs** | After preprocessing | | 4 659 | | 8 187 | |
|  | Rarefied | 1 000 | 3 369 | **72%** | 3 132 | **38%** |
|  |  | 10 000 | 3 317 | **71%** | 6 590 | **80%** |

**Supplementary Table S2.** Alpha-diversity differences statistical testing using Wilcoxon test. The test values (W) and FDR-adjusted *P*-values are given.

| **Dataset** | **Index** | **Comparison** | | | | **W** | ***P*** |
| --- | --- | --- | --- | --- | --- | --- | --- |
|  |  | **Group 1** | **N** | **Group 2** | **N** |  |  |
| **PRJNA930592** | Inverse Simpson | bee | 165 | honey | 405 | 38395 | 0.008 |
|  |  | bee | 165 | plant | 221 | 23194.5 | < 0.001 |
|  |  | honey | 405 | plant | 221 | 47395 | 0.222 |
|  | Shannon | bee | 165 | honey | 405 | 33331 | 0.964 |
|  |  | bee | 165 | plant | 221 | 22616 | < 0.001 |
|  |  | honey | 405 | plant | 221 | 53076 | < 0.001 |
| **PRJEB31847** | Inverse Simpson | seed | 151 | *Apis* | 10 | 350 | 0.015 |
|  |  | seed | 151 | nectar | 24 | 1462 | 0.185 |
|  |  | seed | 151 | pollen | 7 | 530 | 0.993 |
|  |  | seed | 151 | *Osmia* | 6 | 139 | 0.015 |
|  |  | *Apis* | 10 | nectar | 24 | 173 | 0.092 |
|  |  | *Apis* | 10 | pollen | 7 | 56 | 0.083 |
|  |  | *Apis* | 10 | *Osmia* | 6 | 32 | 0.875 |
|  |  | nectar | 24 | pollen | 7 | 97 | 0.704 |
|  |  | nectar | 24 | *Osmia* | 6 | 34 | 0.083 |
|  |  | pollen | 7 | *Osmia* | 6 | 2 | 0.023 |
|  | Shannon | seed | 151 | *Apis* | 10 | 364 | 0.016 |
|  |  | seed | 151 | nectar | 24 | 1511 | 0.241 |
|  |  | seed | 151 | pollen | 7 | 640 | 0.387 |
|  |  | seed | 151 | *Osmia* | 6 | 140 | 0.015 |
|  |  | *Apis* | 10 | nectar | 24 | 170 | 0.101 |
|  |  | *Apis* | 10 | pollen | 7 | 56 | 0.083 |
|  |  | *Apis* | 10 | *Osmia* | 6 | 28 | 0.875 |
|  |  | nectar | 24 | pollen | 7 | 105 | 0.487 |
|  |  | nectar | 24 | *Osmia* | 6 | 32 | 0.083 |
|  |  | pollen | 7 | *Osmia* | 6 | 2 | 0.023 |

**Supplementary Table S3.** Beta-diversity differences significance testing using environment fit after the NMDS ordination. The freedom degrees (df), test values (*F*), significance criterion (Pr(*>F*)), and predicted R^2^ values are given. In the fields model and variable, abbreviation env is used for the environment (grouped environments - classes in the analysis). There is the subgroup env[bees] - analysis performed for the differences between *Apis* and *Osmia* communities.

| **Dataset** | **Model** | **Index** | **Variable** | **df** | **R^2^** | ***P*** |
| --- | --- | --- | --- | --- | --- | --- |
| **PRJNA930592** | ~ env + year + mon | Bray-Curtis | env | 6 | 0.071 | 0.001 |
|  |  |  | year |  | 0.187 | 0.001 |
|  |  |  | month |  | 0.001 | 0.001 |
| **PRJEB31847** | ~ env[full] |  | env | 5 | 0.476 | 0.001 |
|  | ~ env[bees] |  | env | 1 | 0.473 | 0.002 |

**Supplementary Table S4.** Beta-diversity differences significance testing using PerMANOVA. The freedom degrees (df), test values (*F*), significance criterion (Pr(*>F*)), and predicted R^2^ values are given. In the fields model and variable, abbreviation env is used for the environment (grouped environments - classes in the analysis). There is the subgroup env[bees] - analysis performed for the differences between *Apis* and *Osmia* communities.

| **Dataset** | **Model** | **Index** | **Variable** | **df** | **R^2^** | ***F*** | **Pr(*>F*)** |
| --- | --- | --- | --- | --- | --- | --- | --- |
| **PRJNA930592** | ~ env + year + mon | UniFrac | env | 2 | 0.088 | 39.2 | 0.001 |
|  |  |  | year | 1 | 0.015 | 13.4 | 0.001 |
|  |  |  | month | 2 | 0.010 | 4.8 | 0.001 |
|  |  | Jaccard | env | 2 | 0.065 | 28.3 | 0.001 |
|  |  |  | year | 1 | 0.018 | 16.2 | 0.001 |
|  |  |  | month | 2 | 0.007 | 3.4 | 0.001 |
|  | ~ env | UniFrac | env | 2 | 0.066 | 27.6 | 0.001 |
|  |  | Jaccard | env | 2 | 0.089 | 38.3 | 0.001 |
| **PRJEB31847** | ~ env[full] | UniFrac | env | 5 | 0.074 | 3.1 | 0.001 |
|  |  | Jaccard | env | 5 | 0.313 | 17.5 | 0.001 |
|  | ~ env[bees] | UniFrac | env | 1 | 0.136 | 2.2 | 0.014 |
|  |  | Jaccard | env | 1 | 0.184 | 3.2 | 0.001 |

**Supplementary Figure S1.** Presented datasets class balance. Column colours are related to the different data groups, and numbers above present samples amounts.


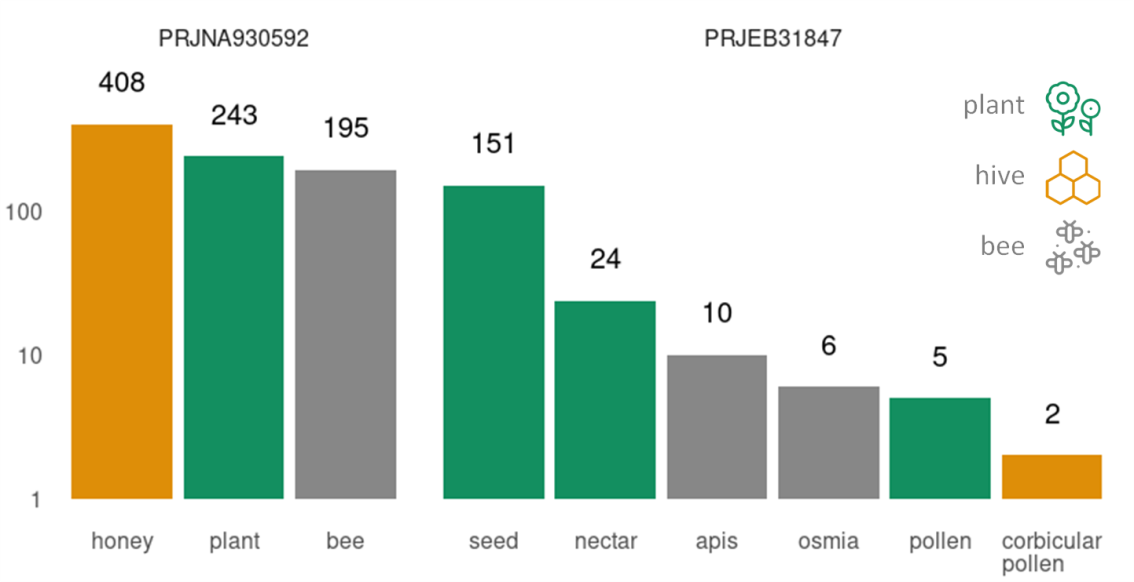


**Supplementary Figure S2.** Non-metric multidimensional scaling (NMDS) plots based on Bray-Curtis dissimilarities for the **(A)** PRJNA930592 and **(B)** PRJEB31847 datasets. Points are colored by the hive of origin or experimental environment and shaped by the source material. Ellipses represent the 95% confidence interval for each group. The group of diverged honey samples is denoted by an asterisk (*).


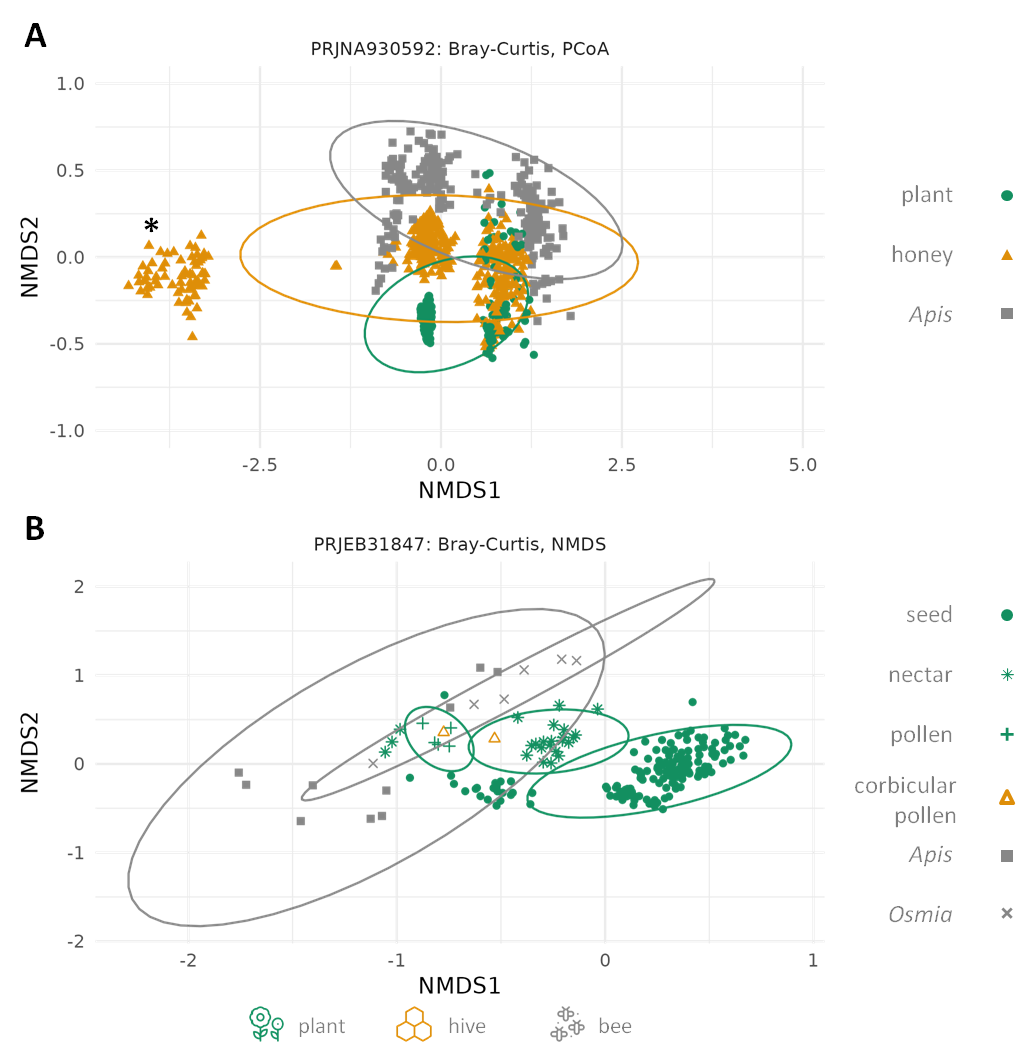


**Supplementary Figure S3.** Influence of *Apilactobacillus kunkeei* abundance on the community diversity in honey samples from the PRJNA930592 dataset after rarefaction to 1,000 sequences per sample. Samples are colored: (AC points) by the abundance of *A. kunkeei*; (B points, C ribbons) by their beta-diversity-based group (where 0* corresponds to the group indicated on Supplementary Fig. S2) and the abundance of *A. kunkeei* (0, 1-250, or > 250 reads). **(A)** Density distribution of *A. kunkeei* read counts. **(B)** Relationship between *A. kunkeei* abundance and sample alpha-diversity (Shannon and Inverse Simpson indices). **(C)** Raincloud plots of alpha-diversity indices. Brackets indicate significant differences based on Wilcoxon rank-sum tests, with corresponding p-values.


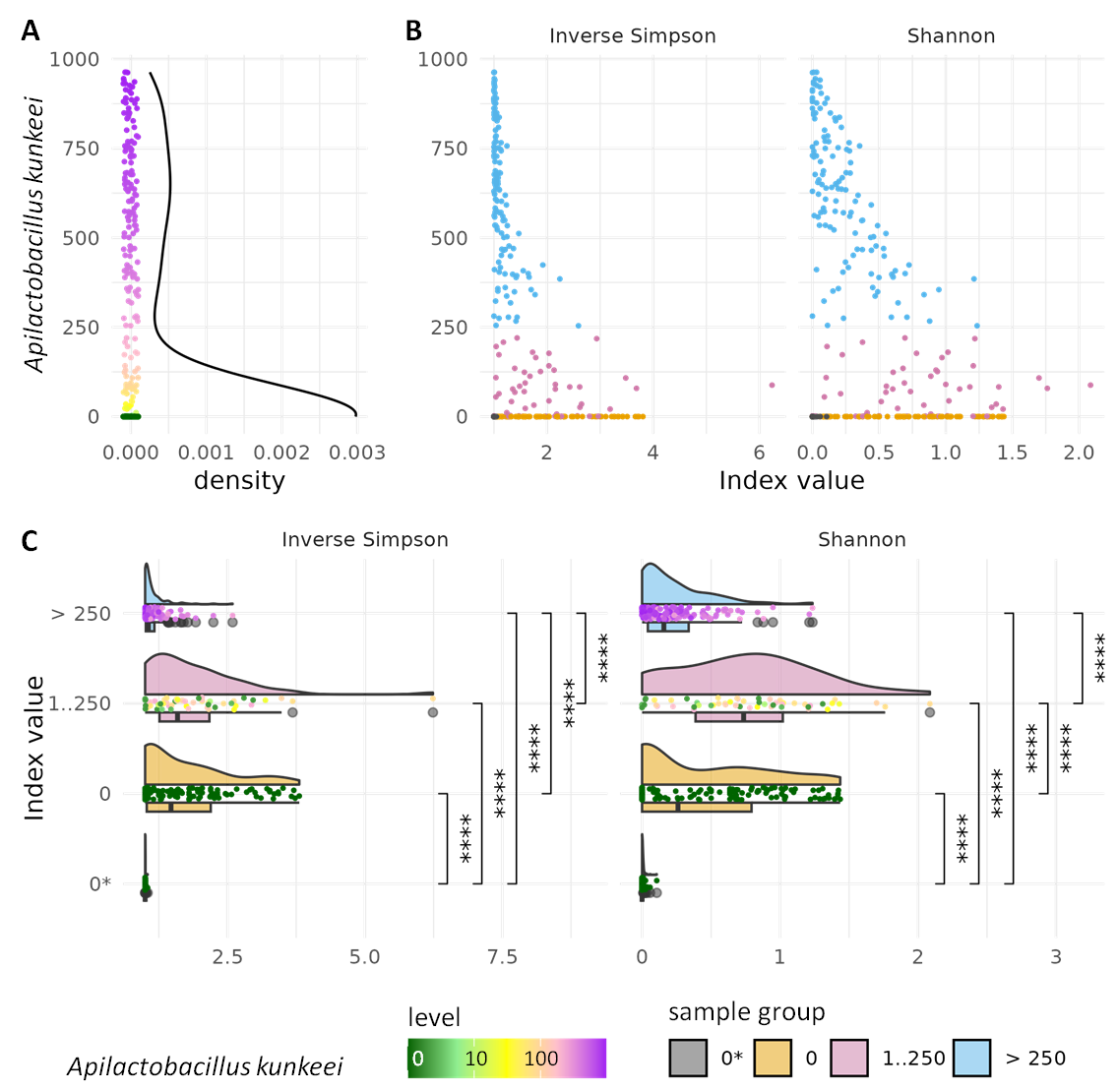


**Supplementary Figure S4.** Overlap of generalist zOTUs between bioprojects. **(A)** Venn diagram identifying zOTUs shared between the PRJNA930592 and PRJEB31847 bioprojects. The analysis includes zOTUs detected across all summarized environments (PRJNA930592: bees, plants, hive; PRJEB31847: bees (*Apis* and/or *Osmia*), plants (nectar, pollen and/or seeds). **(B, C)** Taxonomic classification at the species level for cosmopolitan zOTUs unique to each bioproject. Panel B shows species shared across honey, bee, and plant samples in PRJNA930592 but absent from one or more environments in PRJEB31847. Panel C shows species shared across plant and bee samples in PRJEB31847 but absent from one or more environments in PRJNA930592.

**
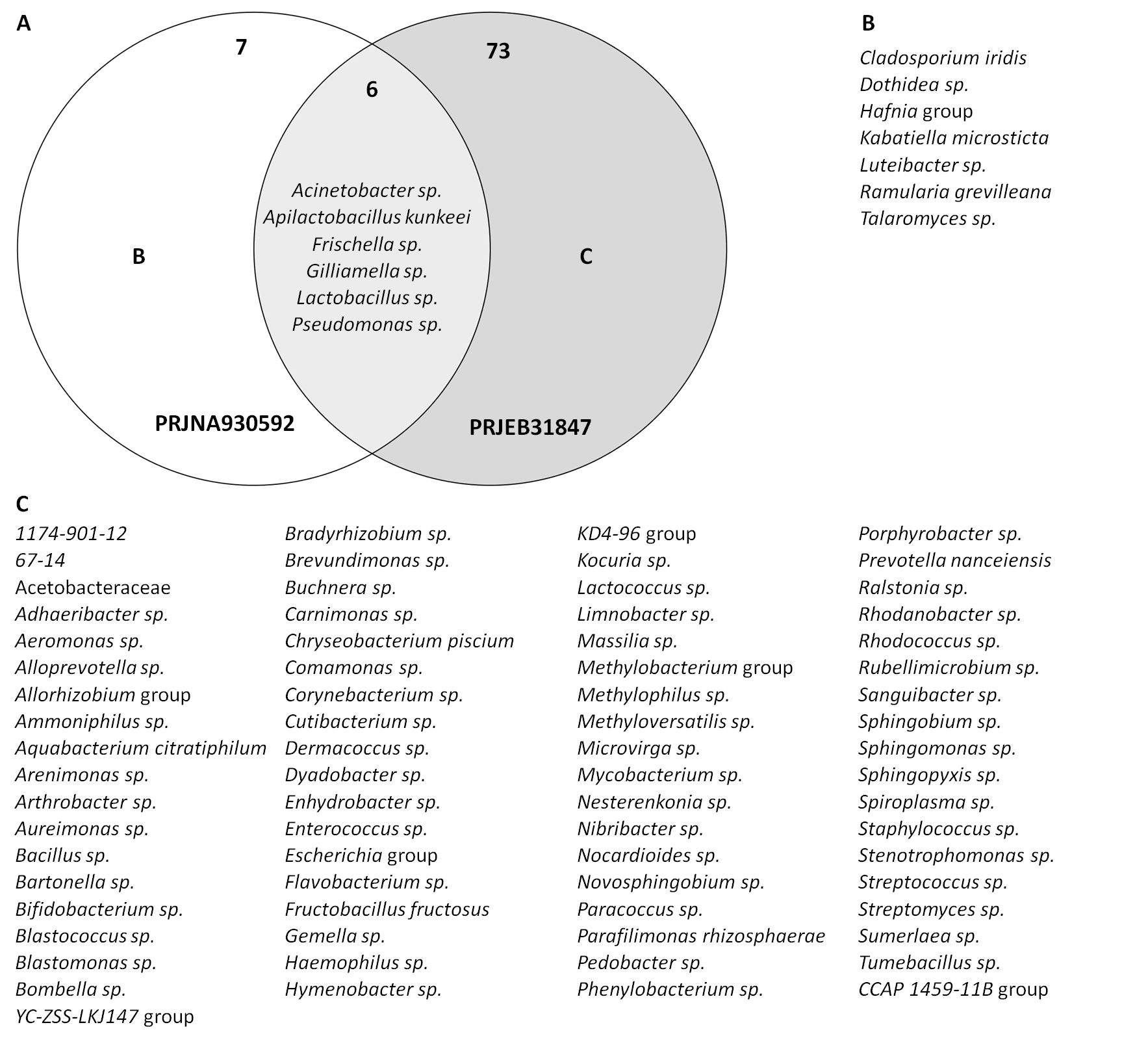
**

**Supplementary Figure S5.** Structural equation modeling (SEM) of microbial transfer pathways in the PRJNA930592 dataset (*Apis mellifera* bees, in-hive *A. mellifera* honey and different plants, natural environment). **(A)** Visualization of the full SEM model. Path coefficients are denoted as follows: ***h*** (transfer from plants to honey), ***b*** (plants to bees), ***c*** (honey to bees), and ***hc*** (indirect transfer from plants to bees via honey). Indirect paths are indicated by dotted lines. Coefficient ***hc*** was calculated as a SEM part as ***(h*c)*** contribution prediction **(B)** Model selection table, showing path coefficients, degrees of freedom (Df), and Bayesian Information Criterion (BIC) scores. The best-fitting model (mediation), used for downstream analysis, is highlighted. **(C)** Summary of significant path coefficients across different taxonomic levels (zOTU, species, family), indicating the number of features involved in the analysis at each level. The color intensity of the cells corresponds to the coefficient value; red cells indicate statistically insignificant paths (p > 0.01).


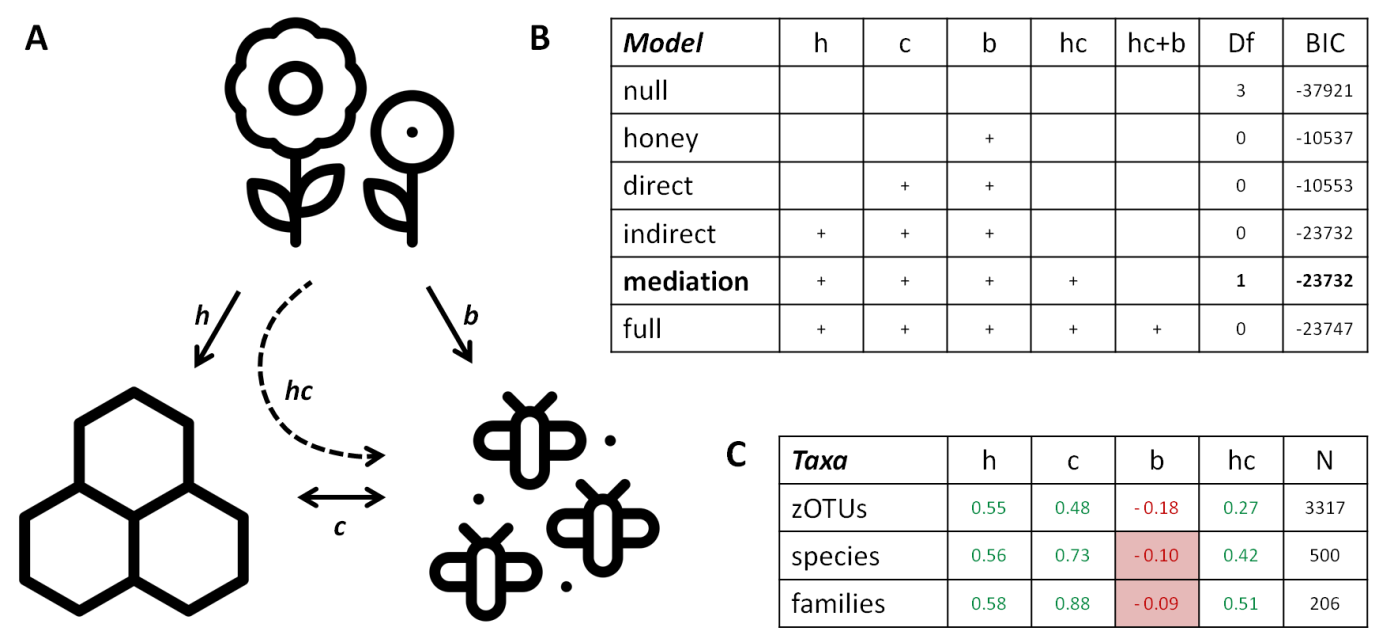


**Supplementary Figure S6.** Structural equation modeling (SEM) of microbial transfer pathways in the PRJEB31847 dataset (*Apis mellifera* and *Osmia bicornis* bees, *Brassica napus* pollen, nectar and seeds, experimental environment). **(A)** Visualization of the full SEM model. Path coefficients are denoted as follows: ***An*** (transfer from nectar to *Apis* bees), ***Ac*** (transfer from corbicular pollen to *Apis* bees), ***As*** (indirect transfer from seeds to *Apis* bees), ***As*** (indirect transfer from seeds to *Apis* bees), ***On*** (transfer from nectar to *Osmia* bees), ***Op*** (transfer from pollen to *Osmia* bees), ***Os*** (indirect transfer from seeds to *Osmia* bees), ***As*** (possible indirect transfer between *Apis* and *Osmia* bees), ***p*** (maturation of pollen), ***ns*** (transfer from nectar to seeds), ***ps*** (transfer from pollen to seeds). Indirect paths are indicated by dotted lines. Indirect coefficients were calculated as contribution prediction as a SEM part (***As*** *:=* ***ps*Ap + ns*An; Os*** *:=* ***ps*Op + ns*On; Acp*** *:=* ***Ac*cp; AO*** *:=* ***An*On + Ap*Op***) **(B)** Model selection table, showing path coefficients, degrees of freedom (Df), and Bayesian Information Criterion (BIC) scores. The best-fitting models, used for downstream analysis, is highlighted. **(C-F)** Summary of significant path coefficients across different taxonomic levels (zOTU, species, family), indicating the number of features involved in the analysis at each level. Non-used data for each model was removed. The color intensity of the cells corresponds to the coefficient value; red cells indicate statistically insignificant paths (p > 0.01). Models: (C) “*plant*”, (D) “*Apis*”, (E) “*Osmia*”, (F) “no cp”.


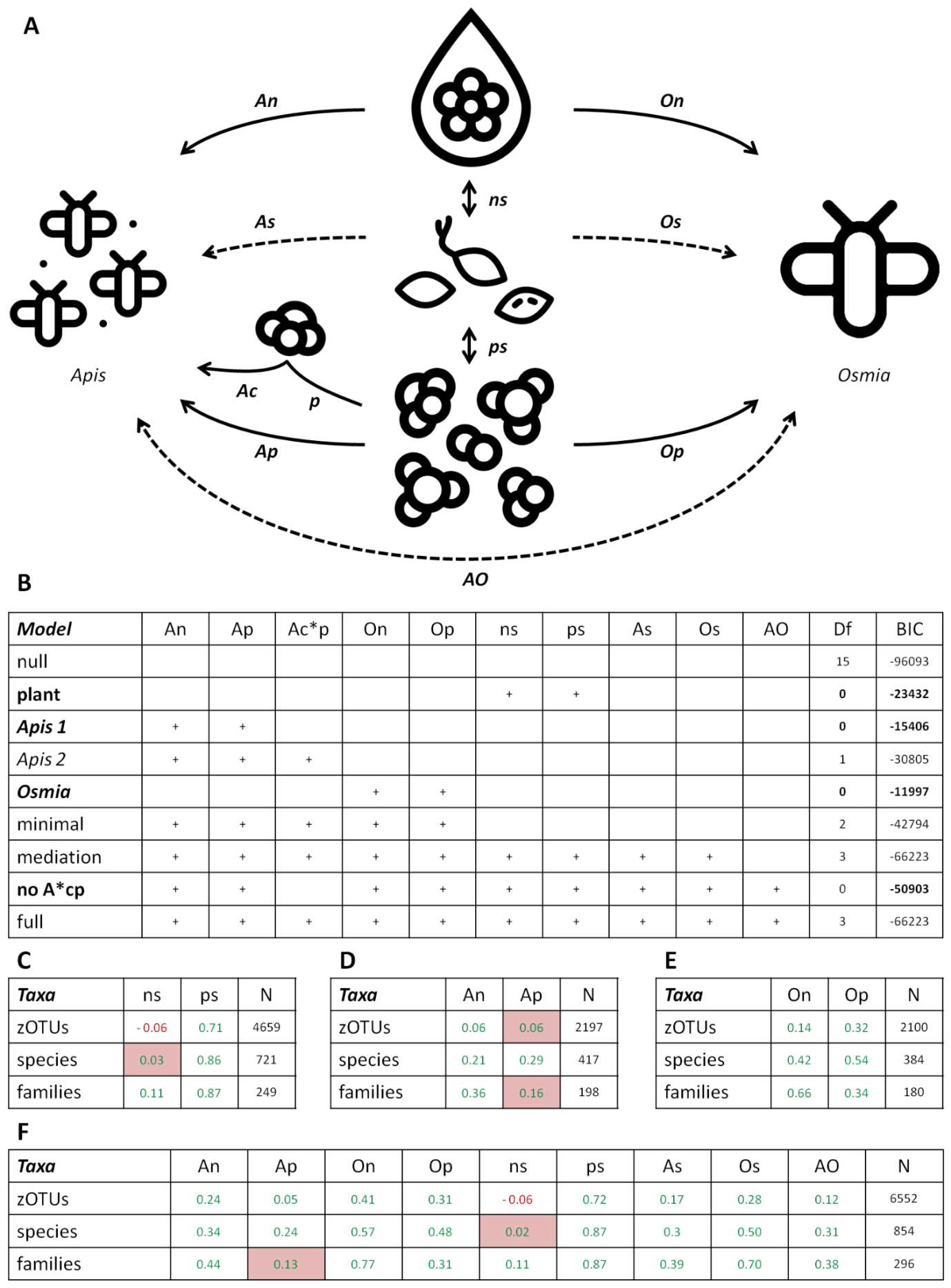

Supplement: ieaf093_Supplementary_Data [file ieaf093_supplementary_data.zip]
